# Comparative In Vitro Study of the Bond Strength of Composite to Carbon Fiber Versus Ceramic to Cobalt–Chromium Alloys Frameworks for Fixed Dental Prostheses

**DOI:** 10.3390/ma13143173

**Published:** 2020-07-16

**Authors:** Rocio Cascos-Sanchez, Pedro Molinero-Mourelle, Rocio Ortega, Ruben Agustin-Panadero, Jaime Del Rio Highsmith, Miguel Gomez-Polo

**Affiliations:** 1 Department of Conservative Dentistry and Orofacial Prosthetics, Faculty of Dentistry, Complutense University of Madrid, 28040 Madrid, Spain; rcascos@ucm.es (R.C.-S.); jrh@odon.ucm.es (J.D.R.H.); mgomezpo@ucm.es (M.G.-P.); 2 Department of Prosthetic Dentistry, Faculty of Dentistry, European University of Madrid, 28670 Madrid, Spain; rocio.ortega@universidadeuropea.es; 3 Department of Stomatology, Faculty of Medicine and Dentistry, University of Valencia, 46010 Valencia, Spain; ruben.agustin@uv.es

**Keywords:** bond strength, Co–Cr alloy, carbon fiber, metal ceramic, fiber-reinforced composite, adhesion, three-point bend test, flexural properties

## Abstract

Purpose: The aim of this comparative in vitro study was to assess the bond strength and mechanical failure of carbon-fiber-reinforced composites against cobalt–chrome structures with ceramic veneering. Materials and methods: A total of 24 specimens (12 per group) simulating dental prosthetic frameworks were fabricated. The experimental specimens were subjected to a thermocycling aging process and to evaluate bond strength. All specimens were subjected to a three-point bending test to fracture using a universal testing machine. Results: The cobalt–chrome/ceramic group yielded a bond strength value of 21.71 ± 2.16 MPa, while the carbon-fiber-reinforced composite group showed 14.50 ± 3.50 MPa. The failure assessment reported statistical significance between groups. Although carbon-fiber-reinforced composite group showed lower bond strength values, the chipping incidence in this group was as well lower. Conclusions: The chrome–cobalt/ceramic group showed greater bonding strength compared to the carbon-fiber-reinforced composite; most of the fractures within the cobalt–chrome/ceramic group, had no possibility of direct clinical repair.

## 1. Introduction

Computer-aided design/computer-aided manufacturing (CAD/CAM) technology in dental medicine has allowed for the development of new biomaterials with the manufacturing of homogeneous prosthetic structures. Carbon-fiber-reinforced composite (CFRC) is a highly biocompatible material composed by 99.9% chemically pure carbon filaments with diameters of 5–10 µm, embedded in an epoxy resin matrix producing braided meshes with 5000 to 8000 fibers [1]. This is about 58% of its weight and 47% of its volume. This composition provides excellent mechanical properties, such as high resistance to fatigue, fracture, abrasion and temperature. Moreover, it presents a low specific weight, thermal expansion coefficient, electrical conductivity and a good buffering of vibration forces [1,2,3,4,5,6]. CFRC has been mainly used in fixed dental prostheses (FDPs) and framework manufacturing [7,8].

In implant prosthodontics, the lack of mechanoreception and proprioception of partially and edentulous ridges implies that CFRC may be particularly favorable for these indications due to its good biomechanical capacity to absorb occlusal forces [1,9].

Compared to metal alloys, this material has advantages, such as, low specific weight, better flexural strength and an excellent chemical adhesion to the veneering resin [1,2,3,4,5,6]. Furthermore, CFRC could be presents in better biomechanical properties in terms of bone resorption around implants compared to metal frameworks, due its elastic properties comparable to dentin [1,9,10].

Despite the recent developments on CAD/CAM materials, metal-based restorations are still considered the gold standard for implant-supported fixed restorations due to their high mechanical resistance and the excellent clinical performance in the medium- and long-term follow-up [11,12,13,14].

Over the last 20 years, with the increase of the price of gold, cobalt–chrome (Co–Cr) alloy has gained importance in metal–ceramic restorations because of its excellent mechanical properties, durability, affordability, absence of nickel, and resistance to corrosion [15,16,17,18].

Although Co–Cr-implant-supported restorations present high success rates, they are not exempt from complications, being one of the most frequent ceramic chipping [19,20,21,22,23,24,25]. The bond strength between the ceramic veneering and the underlying metal framework is a key factor linked to the success and survival of these restorations [26,27,28]. In a preclinical scenario, several methods have been proposed to determine the bond strength between materials, being one of the most used the 3-point bend test [29,30].

Considering technical complications, the use of CRFC may present advantages over conventional restorations, where the chemical and mechanical bonding of organic components increases the bonding strength between the two materials, reducing the possibility of chipping.

The black color aspect of the CRFC is one of the disadvantages that this material presents. However, it can be covered using opaque layers masking the surface of prosthetic structures with good results [31] due to the optical properties that provide composite resins and the variety of dyes, intensities, values, existing opacities and translucencies.

Therefore, the aim of this in vitro study was to assess and compare the bond strength and the fracture behavior between metal–ceramic frameworks and CRFC frameworks. The first null hypothesis was that there would be no difference between bond strength among groups. The second null hypothesis was that the materials tested did not have an influence on the fracture type.

## 2. Materials and Methods

A sample size of 12 was calculated for each group, determined according to similar studies [28,29,30,31,32]. The standard deviation (S) was set at 4.40 and the alpha error (E) at 1.90. It was based on the following formula:

$$N=\frac{t_{N-1}^{2}\times S^{2}}{E^{2}}$$

A total of 24 specimens were made: 12 cobalt–chrome structures with ceramic veneering (Co–Cr) and 12 carbon-fiber structures veneered with composite resin (CFRC).

All the studied specimens were designed and manufactured according to UNE-EN ISO 178:2010 standards [33], following the manufacturer’s specifications and by the same dental technician. A digital design of the Co–Cr and CFRC frameworks was created using computer software (Dental System^®^ 3 Shape Designing Software^®^ V.2017, 3Shape, Copenhagen, Denmark) with dimensions of 35 × 10 × 2 mm. The generated Standard Tesselaction Language (STL) file was processed by a 5-axis milling machine (S2, VHF Camfacture AG^®^, Ammerbuch, Germany), Table 1 and the specimens were made from Co–Cr (Easy Disc, Starbond^®^, S&S Scheftner GmbH, Mainz, Germany) and CFRC (Bio carbon tablet, MICRO MEDICA SRL^®^, Robbio, Italy) CAD/CAM discs. Once the framework manufacturing had finished, all specimens were evaluated for the required 2-mm-thickness using a digital caliper (DC01 Digital Caliper Carbon Fiber, TackLife, Shenzhen, China)

### 2.1. Specimen Fabrication

#### 2.1.1. Co–Cr Specimen Processing

The Co–Cr specimens were sandblasted with 110 μm aluminum oxide (Al_2_O_3_), particles (Basic Classic, Renfert^®^ GmbH, Hilzingen, Germany) for 15 s at a pressure of 200 kPa at a distance of 10 mm and were then cleaned ultrasonically for 5 min. The specimens were then placed in a dental ceramic furnace (Austromat 624 Oral Design, Dekema^®^, Freilassing, Germany) for degassing and oxidation treatment at 990 °C before the ceramic application. A bonding agent (SR Link 5 ml, Ivoclar Vivadent^®^, Schaan, Liechtenstein), opaquer, four layers of body ceramic (E.max Ceram, Ivoclar Vivadent^®^, Schaan, Liechtenstein) and glazing layer were applied followed the recommendations of the manufacturer to achieve a total thickness of 2 mm. All specimens were fired 5 times during the veneering process in a ceramic furnace (Austromat 624 Oral Design, Dekema^®^, Freilassing, Germany). Table 2.

#### 2.1.2. CFRC Specimens Processing

The CFRC specimens’ group were first sandblasted with 110 μm aluminum oxide particles (Renfert^®^, Hilzingen, Germany) for 15 s with a pressure of 200 kPa at a 10 mm distance and were cleaned ultrasonically for 5 min. A bonding agent (BioXfill, Micromedica SRL^®^, Robbio, Italy) was applied on all specimens, followed by an opaquer layer and four resin composite layers (1 dentin layer, 1 translucent layer and 2 incisal layers) (SR Nexco Paste, Ivoclar Vivadent^®^, Schaan, Liechtenstein) to obtain a thickness of 2 mm. All layers were light-cured (HiLite power 3D, Kulzer^®^ GmbH, Hanau, Germany) for 180 s following the manufacturer’s recommendations. Subsequently, all specimens from the CFRC group were polished using universal polishing paste (Ivoclar Vivadent^®^, Schaan, Liechtenstein) in the polisher (R-080160, Mestra^®^, Talleres mestraitúa, S.L., Bilbao, Spain).

After their manufacture, the specimens were measured in their length, width and thickness by a digital caliper (Tacklife-DC01, Kinnek Business Solutions LLC, New York, NY, USA), to ensure their standardization. To verify the thickness, three points, the two ends and the central area were measured. A discrepancy of ±0.2 mm was allowed in relation to the thickness of the specimens.

### 2.2. Thermocycling

The experimental groups were subjected to a 10,000-cycle thermocycling process according to ISO TS 11405 standards [35] in distilled water at 5 °C and 55 °C, for 20 s with a 10-s dwell time (VA55, EuroOrtodoncia, Madrid, Spain). After the thermocycling process, all specimens were examined under an optical microscope (Telecentric Objective 1:1, Jos. Schneider Optische Werke GmbH, Bad Kreuznach, Germany) at 4× magnification Toupview V.x643.7.6701 (Toupview, ToupTek, ToupTek Photonics Co., Ltd, Hangzhou, China) to verify that there were no alterations in the frameworks or veneering material.

### 2.3. Mechanical Testing

**Figure 1:**
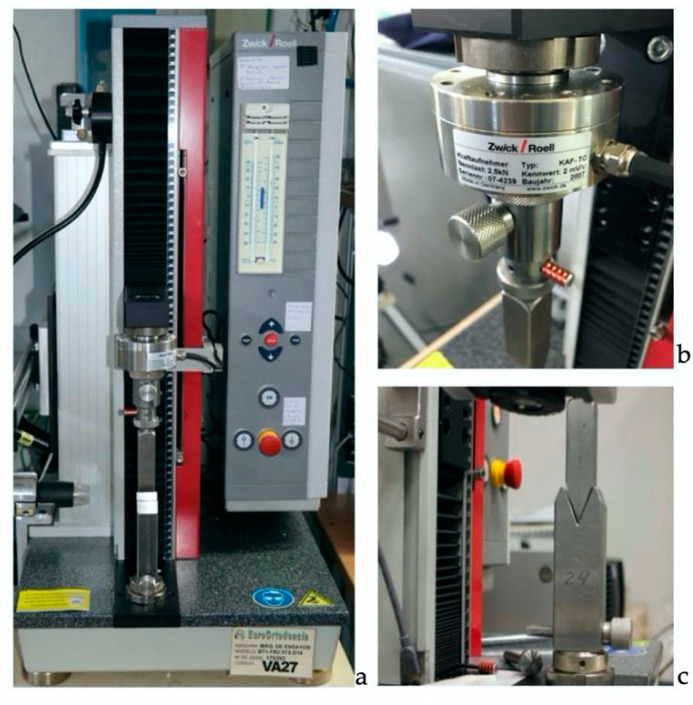
(**a**) Universal testing machine. The machine consists of (**b**) a load cell, in charge of measuring pressure and traction; and a motor, in charge of placing the cell at the appropriate distance. In addition, it consists of the (**c**) supports in charge of simulating 3-point bend test.

All specimens were subjected to a three-point bending test to fracture using a universal testing machine (Zwick/Roell BT1FR2. 5TS.D14, 179392, VA27; Zwick/Roel, Ulm, Germany) in accordance with ISO 14125:1998 [36] and ISO 178:2010 standards [33] (Figure 1).

For the test, a holder with a distance between its supports of 32 mm was used in accordance with the ISO 178:2010 standard [33]. A preload of 0.2 N was established at an E-module speed and a test speed of 1 mm/min, in environmental conditions of 20 °C. A constant force with a speed of 1 mm/min was applied in the center of the specimen up to a maximum force of 2500 N (at an equidistant point between the supports) or a maximum of 10-mm deformation.

Strain–stress curves and a magnification video were registered for all the samples during the bending test by using testXpert^®^II V143 software (Zwick/Roell, Ulm, Germany) and obtained data were recorded.

### 2.4. Failure Assessment

All the specimens were evaluated by using naked-eye assessment, tactile-probe assessment and under the optical microscope at 4× resolution, (4912 × 3684 pixels) to determine the fracture type as follows: adhesive (between the framework and covering material), cohesive (entirely within the framework or coating material) or mixed (a combination of adhesive and cohesive failure) [27,28,29,30,31,32,37].

### 2.5. Data Analysis

Statistical analysis was performed using IBM-SPSS-22 software (SPSS Statistics v 22.0, IBM Corp^®^, Armonk, NY, USA). The mean values and standard deviations (SD) of the results were determined and to compare the goodness of fit to the normal distribution of the variables, the Shapiro–Wilk test was performed. The quantitative and qualitative variables were statistically analyzed using the Mann–Whitney U test and the chi-squared test (X^2^), respectively. The statistical significance was established at ≤ 0.05, except in the Shapiro–Wilk test that was established as α = 0.01. *p*-values of less than 0.5 were considered as statistically significant differences, except in the Shapiro–Wilk test, being *p* = 0.01.

## 3. Results

**Figure 2:**
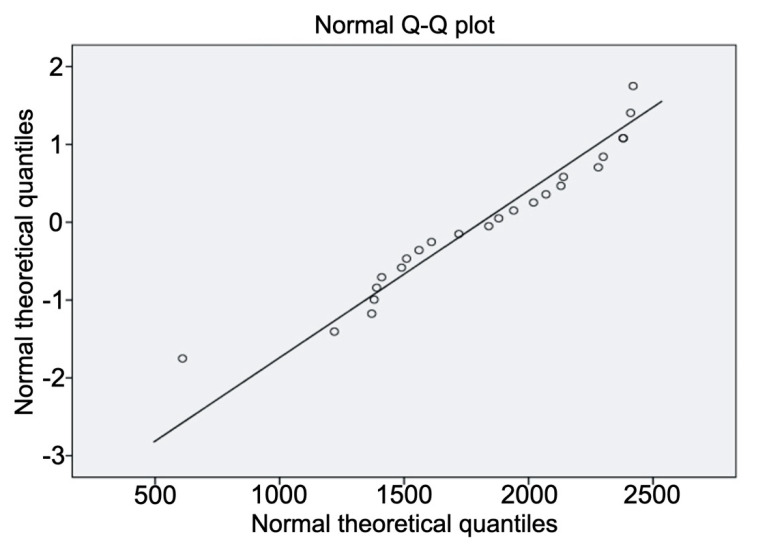
Representative Q–Q plot for bond strength confirming the trend towards normality of the sample.

Using Q–Q graphs of normality (Figure 2) and the Shapiro–Wilk test, it was verified that the data fit the Gauss model of normality (*p* > 0.05). The statistical normality fit was confirmed, which allowed the use of a parametric test as the statistical method of comparison between groups. All the studied variables were sufficiently close to the Gaussian normality model.

**Figure 3:**
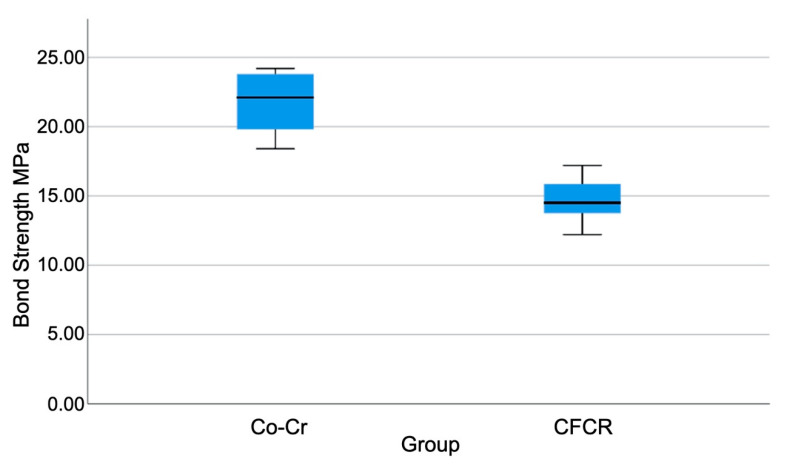
Representative box plot for bond strength according to the studied materials.

Statistically significant differences (*p* < 0.01) were observed in the bond strength values following the Mann–Whitney U test, obtaining higher values in the Co–Cr control group (21.71 ± 2.16 MPa) against the CFRC test group (14.50 ± 3.50 MPa). The highest value (24.20 MPa) was found in the Co–Cr group while the lowest value (6.10 MPa) was observed in the CFRC group (Figure 3).

**Figure 4:**
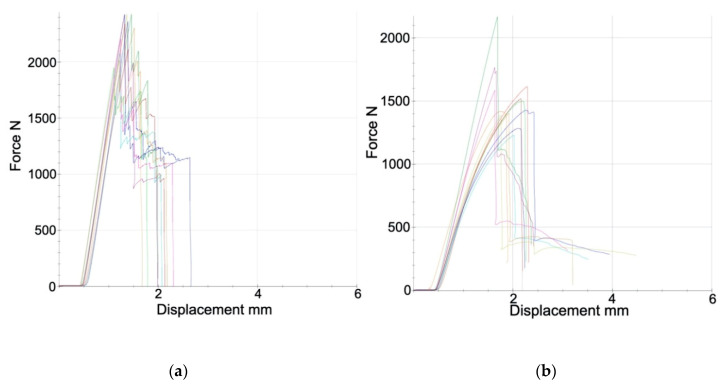
Force–displacement diagrams. (**a**) Cobalt–chromium frameworks with ceramic veneering (Co–Cr), (**b**) carbon-fiber framework with resin veneering (CFRC).

Force–displacement diagrams show the mechanical behavior of the groups (Figure 4). The CFRC curves show a parabolic trend deviating from the straight line, indicating an elastic deformation of the material. The Co–Cr group showed a linear curve trend, indicating a nonlinear elasticity of the material and consequent plastic deformation.

**Figure 5:**
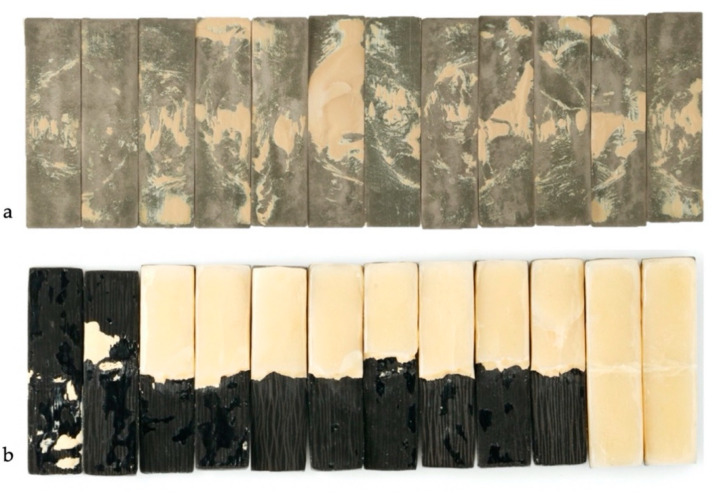
Specimen surface after fracture failures. (**a**) Metal–ceramic specimen failures; (**b**) carbon-fiber-reinforced composite specimen failure.

**Figure 6:**
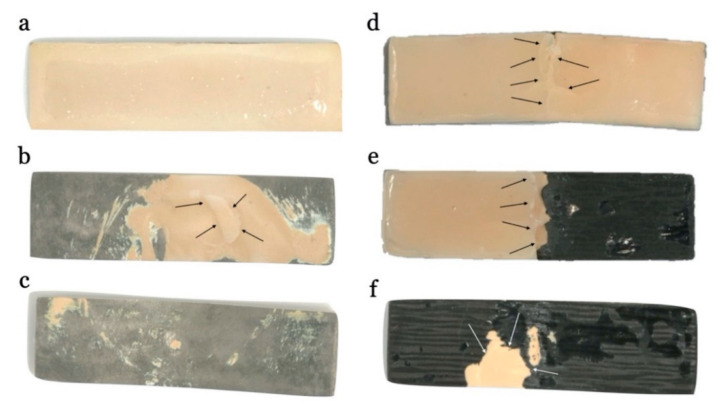
Failure analysis of (**a**) metal–ceramic specimen before testing; (**b**) mixed fracture in metal–ceramic specimen; (**c**) adhesive-type fracture in the metal–ceramic specimen, a complete chipping from the Co–Cr structure occurs; (**d**) cohesive fracture in carbon-fiber reinforced composite specimen, fracture only occurs in the internal layer of the composite resin; (**e**) mixed fracture in carbon-fiber reinforced composite specimen; (**f**) adhesive fracture in carbon-fiber reinforced composite specimen, a complete chipping of the coating material from the carbon-fiber framework occurs.

The results according to the type of fracture following the chi-squared test (Table 1), revealed a high statistical difference (*p* < 0.01) and a 57% of effect between the Co–Cr group and the CFRC group. Adhesive failure with fully ceramic veneering chipping was reported in 91.7% of the Co–Cr structures and the remaining failures within this group were of a mixed type (8.3%), (Table 3, Figure 5 and Figure 6). On the other hand, 66.7% of the CFCR specimens presented a mixed-type failure, preserving the resin coating to fiber framework. The rest of the failures in this group were cohesive-type fractures in 16.7% of the specimens and adhesive in the remaining 16.7% (Figure 6).

## 4. Discussion

The literature presents an obvious scarcity of in vitro clinical trials of metal-free restorations, particularly trials of those materials that claim to introduce innovative advantages to the field of prosthetic dentistry. The present study used metal–ceramic restorations as its control group, as according to the literature is the most supported [18,19,20,21].

The bending testing used in the present trial has been described as the most effective means of evaluating the fracture resistance of restorations and has been used by various authors in the literature [26,27,28,29,30,31,32].

Several methods have been used to test bond strength; however, no specific standardized method has been reported to measure this parameter accurately [30,37,38,39,40,41,42,43,44,45]. Moreover, the methodological heterogeneity reported in the literature in terms of the geometric forms of testing, as well as in the distribution of forces, result in differences in the bond strengths obtained among studies [41,42,43,44,45].

Nevertheless, the ANSI/ADA specification No. 38 and ISO 9693 [46] recommend the use of a three-point bending test to evaluate the strength of the metal–ceramic bonding in the same way as ISO 14125:1998 [36] for fiber-reinforced plastic compounds [26,32,45,47]. The 3-point bending test best simulates clinical scenarios; therefore, it was the test of choice in this study to evaluate the bonding strength between the groups.

The purpose of this in vitro study was to compare the bond strength and fracture behavior between Co–Cr with ceramic veneering and CFCR. Considering the obtained results, the first null hypothesis was rejected, as statistically significant differences in the bond strength between the Co–Cr and CFRC groups were revealed. Likewise, the second null hypothesis was also rejected, as significant differences were observed for the failure types analysis between Co–Cr and CFRC.

Although the Co–Cr specimens revealed a higher bonding strength than the CFRC group, these results differ from other studies that reported higher values in metal–ceramic specimens [38,39,47,48,49].

On the other hand, to the best of our knowledge, no previous studies have reported shear bond strength testing values for CFRC. Therefore, these results cannot be directly compared due the lack of homogeneity in methodology [50].

Among the factors that determine bonding strength between surfaces, it has been reported that they could depend on the primary components of the materials [30,51]. For metal–ceramic restorations, bonding is affected by the composition and the thickness of the oxide film that forms on the metal surface. When this layer is absent, thin or thick, the resulting bond strength will be weak [30,37,38,39,41,52,53]. It has been reported that the morphology of the oxidation layer will depend on the techniques used in the manufacturing of Co–Cr frameworks [39,53,54]. Few preclinical or clinical studies evaluating the behavior of carbon fiber and composite exist, however, the same organic nature of its components has been reported and theoretically presents a better chemical bond. On the other hand, in terms of mechanical retention, sandblasting with aluminum oxide particles is used to increase the contact surface between materials, thus improving mechanical retention and wettability [30,49].

The particle size and material for particle abrasion has been the outcome studied by some authors, who have decided to maintain the size at 110 μm in accordance with the methodology of this study [49].

No literature has been found on the mechanical bond between CFRC and composite resin either. The fractures that occur between two materials could be classified as adhesive, cohesive and mixed fractures [30,55]. A cohesive failure indicates that the bond between the framework material and the overlay material is stronger than both materials independently and therefore more force will need to be applied to separate these materials [39,41].

As shown in Figure 2, visual and optical microscopy results of the debonding surfaces reveal a direct relationship between the failure type and the different groups studied. The Co–Cr group presented adhesive failures in 91.7% of the specimens, producing leap-type fractures of the ceramic until the total chipping. This failure between the Co–Cr framework interface and the ceramic veneering could be influenced by the physicochemical properties of both materials, by the mechanical preparation of the surface as well as by the properties of the applied adhesive [56].

These results are not consistent with those reported in the literature, where the type of fractures in metal–ceramic restorations are described when comparing the metal surface treatments [37] or different fabrication techniques such as casting, milling and selective laser melting [30,38,41,48]. Similarly, mixed fractures, in which 50% or more of the resin coating remained, were predominant in the CFRC group. One of the advantages of these polymeric materials is the ability to be intraoral repair capacity, in contrast to metal–ceramic restorations, which in many cases require the removal of the prosthesis and reveneering. The repairmen process success will depend on the bond strength between the old and new repair compound, the chipping or fracture extension and the surface mechanical preparation. Aging and water absorption lead to degradation of the inhibited oxygen layer, which is necessary for bonding between the two layers [56,57]. To solve this problem, it has been proposed to perform both a mechanical (sandblasting with aluminum oxide, bur abrasion, acid etching and laser irradiation) and a chemical one (application of bonding resin and silane) of the surface to be repaired. Although these techniques have been described, there is currently no consensus on the protocol to follow [58,59,60].

Comparing results regarding bonding strength, metal–ceramic specimens reported better data. These results are not in the same line with those obtained in a similar study by Taufall et al. who described better results in carbon–resin composite fiber specimens as well as in the carbon–resin acrylic fiber. This difference may have been due to the use of different bond strength testing methods [50].

The present study showed the main limitation of in vitro studies, since the obtained data cannot always be clinically extrapolated. The study was innovative, as it proposes a new material for prosthetic restorations. In vitro and in vivo studies are needed to evaluate the behavior of composite resin to carbon-fiber restorations.

Based on the obtained results from this translational study model, future research with a larger sample and new different CAD/CAM materials could provide more power and information to compare. Moreover, clinical studies based on this comparative model for tooth and implant-supported fixed restorations are necessary to be able to refute the data in a clinical human model.

## 5. Conclusions

Based on the findings of this in vitro study the following conclusions were drawn.

1. The cobalt–chrome/ceramic group showed greater bonding strength compared to the carbon-fiber-reinforced composite.
2. While in the carbon-fiber-reinforced composite group, most fractures were mixed or cohesive types, presenting a greater possibility of clinical repair, in the cobalt–chrome/ceramic group fractures, where most had no possibility of direct clinical repair.

## Figures and Tables

**Table 1:** Composition by percent mass of Co–Cr alloy.

Component	Percentage (%)
Co	61%
Cr	27.5%
W	8.5%
Si	1.6%
C, Mn, Fe	<1%

**Table 2:** Firing schedules of veneering procedure for Co–Cr group in accordance with manufacturer and laboratory instructions [34].

Program	Preheating (°C)	Drying Time (min)	Heating Rate (°C/min)	Maximum Temp. (°C)	Total Time (min)
oxidation	650	2	55	990	17
metal bond	550	3	80	980	15
opaquer	550	3	80	930	15
body porcelain	580	3	45	890	17
glaze	600	3	55	890	14

**Table 3:** Inferential analysis: difference between percentages. Percentages of the different types of fracture produced depending on the groups. Adhesive-type fractures predominate in the Co–Cr group with 91.7% compared to 66.7% for the mixed type in the CFRC group.

Failure Type	Co–Cr	CFCR	X^2^	p	R^2^
Cohesive	0.0%	16.7%	13.68 **	0.001	0.570
Adhesive	91.7%	16.7%			
Mixed	8.3%	66.7%			

X^2^—chi-squared frequencies test; p—statistically significant differences (p < 0.05); R^2^—effect size calculation, **: Highly significant p < 0.001.
